# Transcriptional analysis of renal dopamine-mediated Na^+^ homeostasis response to environmental salinity stress in *Scatophagus argus*

**DOI:** 10.1186/s12864-019-5795-x

**Published:** 2019-05-24

**Authors:** Maoliang Su, Jianan Zhou, Zhengyu Duan, Junbin Zhang

**Affiliations:** 1 Shenzhen Key Laboratory of Marine Bioresource & Eco-Environmental Science, College of Life Sciences and Oceanography, Shenzhen University, Shenzhen, 518060 China; 2 Key Laboratory of Optoelectronic Devices and Systems of Ministry of Education and Guangdong Province, College of Optoelectronic Engineering, Shenzhen University, Shenzhen, 518060 China; 3 Key Laboratory of Exploration and Utilization of Aquatic Genetic Resources, Ministry of Education, College of Fisheries and Life Sciences, Shanghai Ocean University, Shanghai, 201306 China

**Keywords:** *Scatophagus argus*, Transcriptome, Salinity stress, Dopamine transport, Na^+^ homeostasis

## Abstract

**Background:**

To control the osmotic pressure in the body, physiological adjustments to salinity fluctuations require the fish to regulate body fluid homeostasis in relation to environmental change via osmoregulation. Previous studies related to osmoregulation were focused primarily on the gill; however, little is known about another organ involved in osmoregulation, the kidney. The salinity adaptation of marine fish involves complex physiological traits, metabolic pathways and molecular and gene networks in osmoregulatory organs. To further explore of the salinity adaptation of marine fish with regard to the role of the kidney, the euryhaline fish *Scatophagus argus* was employed in the present study. Renal expression profiles of *S. argus* at different salinity levels were characterized using RNA-sequencing, and an integrated approach of combining molecular tools with physiological and biochemical techniques was utilized to reveal renal osmoregulatory mechanisms in vivo and in vitro.

**Results:**

*S. argus* renal transcriptomes from the hyposaline stress (0‰, freshwater [FW]), hypersaline stress (50‰, hypersaline water [HW]) and control groups (25‰) were compared to elucidate potential osmoregulatory mechanisms. In total, 19,012 and 36,253 differentially expressed genes (DEGs) were obtained from the FW and HW groups, respectively. Based on the functional classification of DEGs, the renal dopamine system-induced Na^+^ transport was demonstrated to play a fundamental role in osmoregulation. In addition, for the first time in fish, many candidate genes associated with the dopamine system were identified. Furthermore, changes in environmental salinity affected renal dopamine release/reuptake by regulating the expression of genes related to dopamine reuptake (*dat* and *nkaα1*), vesicular traffic-mediated dopamine release (*pink1*, *lrrk2*, *ace* and *apn*), DAT phosphorylation (*CaMKIIα* and *pkcβ*) and internalization (*akt1*). The associated transcriptional regulation ensured appropriate extracellular dopamine abundance in the *S. argus* kidney, and fluctuations in extracellular dopamine produced a direct influence on Na^+^/K^+^-ATPase (NKA) expression and activity, which is associated with Na^+^ homeostasis.

**Conclusions:**

These transcriptomic data provided insight into the molecular basis of renal osmoregulation in *S. argus*. Significantly, the results of this study revealed the mechanism of renal dopamine system-induced Na^+^ transport is essential in fish osmoregulation.

**Electronic supplementary material:**

The online version of this article (10.1186/s12864-019-5795-x) contains supplementary material, which is available to authorized users.

## Background

Salinity is an important environmental factor that significantly affects the physiology of marine fish, influencing their reproduction, survival and distribution [1]. The majority of teleost fish can only endure limited salinity fluctuations after long-term acclimation and, hence, are restricted in their ability to move between different environments [2–4]. Euryhaline fish offer valuable sources for studying osmoregulation as they frequently move from high- to low-salinity areas and must rapidly adapt to such conditions. Thus, these fish present a unique opportunity to study physiological responses in osmoregulatory organs under different salinities. *Scatophagus argus* is a euryhaline fish distributed widely throughout coastal and estuarine habitats in the Indian-Pacific Ocean [5, 6]. Shallow coastal and estuarine waters are characterized by a wide range of salinity fluctuations. Adaptation to this type of environmental variability is primarily achieved via osmoregulation, which is a common trait found in marine animals living in habitats with fluctuating salinity [7]. Previous studies have shown no evidence of abnormal activity or mortality in *S. argus* after abrupt osmoregulatory shock [8–10], implying *S. argus* possesses highly effective osmoregulatory mechanisms that prevent negative effects produced by salinity fluctuations.

Fish in hypersaline water excrete ions through the gill and kidney, and absorb water through the intestine to prevent dehydration. However, in hyposaline water, fish actively ingest ions through their gills to compensate for ion loss and produce dilute urine via reabsorption to protect against passive ion loss, primarily sodium ion (Na^+^) loss, in the kidney [11–13]. Na^+^/K^+^-ATPase (NKA), as an ion-transporting enzyme, is mainly responsible for pumping 3 Na^+^ ions out of the renal proximal tubule cell across the basolateral membrane while pumping two potassium (K^+^) ions into the cell in every pump cycle [14, 15].

The gill and kidney are intricately involved in regulation of ion balance because they are composed of numerous ion channels, pumps and exchangers [16]. However, previous salinity adaptation studies were focused primarily on the gill, and little is known about renal osmoregulation in fish. In mammals, the kidney participates in maintaining body fluid homeostasis, electrolyte concentrations and extracellular fluid volume [17]. The kidney performs homeostatic functions both independently and in concert with other organs, particularly under the control of the endocrine system via paracrine and autocrine pathways [18]. Dopamine, a catecholamine hormone, can be synthesized and secreted in the kidney apart from as a neurotransmitter in the brain [19–22]. Renal dopamine directly inhibits Na^+^ transporters to affect Na^+^ excretion and resorption in the kidney [19]. Activation of the renal dopamine system accounts for at least 50% of Na^+^ excretion during salt loading [23]. By contrast, when sodium intake is low, the ability of renal endogenous dopamine to inhibit Na^+^ transport is abolished. This phenomenon is attributable to the overriding effects of the renin-angiotensin-aldosterone system for salt-conserving mechanisms [24].

Thus far, osmoregulatory studies concerning renal functions in fish are based on findings in mammals. Therefore, relative studies are desperately needed for fish. Among marine fish, adaptation to salinity changes in the kidney is a complex process that requires coordinated activities by numerous osmosensing, signal-transducing, effector and cell-signaling molecules [25]. Marine fish can recognize and respond to salinity variation by altering the expression of genes specifically required for osmoregulation. Osmoregulatory studies at the transcriptomic level can provide new insights into the adaptive responses to salinity changes [26–28]. Previous studies concerning the osmoregulatory mechanisms of fish only involved one or several genes. To better understand the biological processes underlying salinity adaptation in euryhaline fishes such as *S. argus*, exploration of the roles of potential genes and pathways related to osmoregulation based on the comprehensive analysis of transcriptomic profiles, particularly involving endocrine functions, is desperately needed.

In this study, high-throughput RNA sequencing (RNA-Seq) was employed to investigate the renal transcriptome profile of *S. argus* with the goal of identifying osmoregulatory genes in the kidney. The primary goals of this study were as follows: (1) utilize an Illumina paired-end RNA-Seq approach to sequence and de novo assemble the renal transcriptome of *S. argus* after exposure to different salinities, (2) identify candidate functional genes or pathways related to renal osmoregulation and (3) validate the functions of candidate genes involved during salinity tolerance in the endocrine system using in vivo and in vitro molecular and cellular techniques.

## Methods

### Collection, maintenance and treatment of fish and sampling procedure

*S. argus* (21.3 ± 4.6 g) fish were collected from the sea near Zhuhai (~ 25‰ salinity), Guangdong Province, China. Fish were reared for 4 weeks in the tank (1.0 m × 1.0 m × 1.0 m) containing 25‰ seawater (SW). The water temperature was maintained at 27 ± 1 °C. For long-term salinity stress experiments, fish were randomly assigned to one of the following three groups: a hyposaline acclimation (0‰ freshwater [FW]) group (FW; *n* = 15), hypersaline water acclimation (50‰ SW) group (HW, *n* = 15) and control group (25‰ SW, *n* = 15). Fish from the FW and HW groups were transferred from the 25‰ SW tank to FW and 50‰ SW tanks, respectively, where they remained for another 4 weeks. Simultaneously, fish from the control group were transferred from their original 25‰ SW tank to a new SW tank with the same salinity. After 4 weeks, fish were anaesthetized with a bath of 50 mg/L Tricaine methane sulfonate (MS-222, Sigma-Aldrich, St. Louis, MO, USA) and then euthanized by a sharp blow to the head. Fifteen fish in each group were divided into three sets. Each set (five fish) represented one biological replicate. Plasma samples were harvested and kept at 4 °C overnight. Serum was separated from blood cells by centrifugation (4200 g, 10 min) and assayed to determine the dopamine concentration. Fresh kidney tissues from each set were sampled, frozen in liquid nitrogen immediately and then stored at − 80 °C until needed. Animal welfare and experimental procedures were performed in accordance with the Guide for the Care and Use of Laboratory Animals (Ministry of Science and Technology of China, 2006) and were approved by the Animal Ethics Committee of Shenzhen University (Reference No. 2014–162).

### RNA extraction and RNA-Seq library preparation

Total RNA was extracted using TRIzol Reagent (Invitrogen, USA) following the manufacturer’s instructions. RNA was treated with DNase I (Invitrogen, USA) and quantified using a Qubit RNA Assay Kit and Qubit™ 4 Fluorometer (Invitrogen, USA). RNA samples (4 μg) were employed to construct an RNA-Seq library using a NEBNext® Ultra™ RNA Library Prep Kit for Illumina (NEB, USA). The indexed libraries were pooled and sequenced on HiSeq™ 2500 sequencing platform (Illumina, USA) to generate 150-bp paired-end reads.

### Transcriptome sequencing and annotation of transcripts

Raw reads from all three groups were combined and quality-filtered using the Trimmomatic read trimming tool [29]. Reads containing 3′- or 5′-ends with an average quality score of less than 20 in a 4-bp sliding window were trimmed, and reads with an average quality score of less than 10 at the beginning or end were also removed. Any reads shorter than 120 bp were excluded from further assembly. All clean reads were used for reference transcriptome assembly, which was based on Trinity version 2015-09-24 in paired-end mode [30]. To remove any misassembled transcripts, raw sequence reads were mapped to the assembled reference transcriptome using Bowtie 1.1.2 (http://bowtie-bio.sourceforge.net/index.shtml) [31]. Transcript abundance was estimated using RSEM software [32], fragments per kilobase per transcript per million mapped reads (FPKM) values were calculated, and transcripts with an FPKM < 1 were removed. To identify transcriptomic differences among the three salinity stress groups (0‰, 25‰ and 50‰), three de novo assembled transcriptomes were assessed using Trinity software (https://github.com/trinityrnaseq/trinityrnaseq/wiki). To investigate the accuracy of the assembled transcriptomes, raw reads were mapped to the assembled transcriptomes using Bwa-0.7.9a with the BWA-MEM algorithm, and mapping statistics were calculated using SAMtools 0.1.19 (http://samtools.sourceforge.net/samtools.shtml) with the SAMtools flagstat command [33, 34]. To test the saturation level of the assembled transcriptome, 20, 40, 60, 80 and 100 million reads were randomly selected from the raw reads and assembled using Trinity software. Subsequently, the number of transcripts was counted, and a FPKM cutoff value ≥1 was used. The assembled transcripts were annotated using NCBI-BLAST 2.2.30 [35] against the datasets of non-redundant protein sequences (NR) in the National Center for Biotechnology Information (NCBI; http://www.ncbi.nlm.nih.gov) and the manually annotated and reviewed protein sequence database (SWISS-PROT database; http://www.ebi.ac.uk/swissprot/) using a cutoff *E*-value threshold (< 1e-6). The BLAST results were extended to the Functional classifications of Gene Ontology (GO; http://www.geneontology.org/), Clusters of Orthologous Groups of proteins (KOG/COG; http://www.ncbi.nlm.nih.gov/COG) and Pathway Annotation of the Kyoto Encyclopedia of Genes and Genomes (KEGG; http://www.genome.jp/kegg/pathway.html). GO annotation was performed using Blast2GO [36]. The statistical enrichment of unigenes in KEGG pathways was tested using KOBAS software [37].

### Differential expression analysis and functional enrichment analysis

**Fig. 1:**
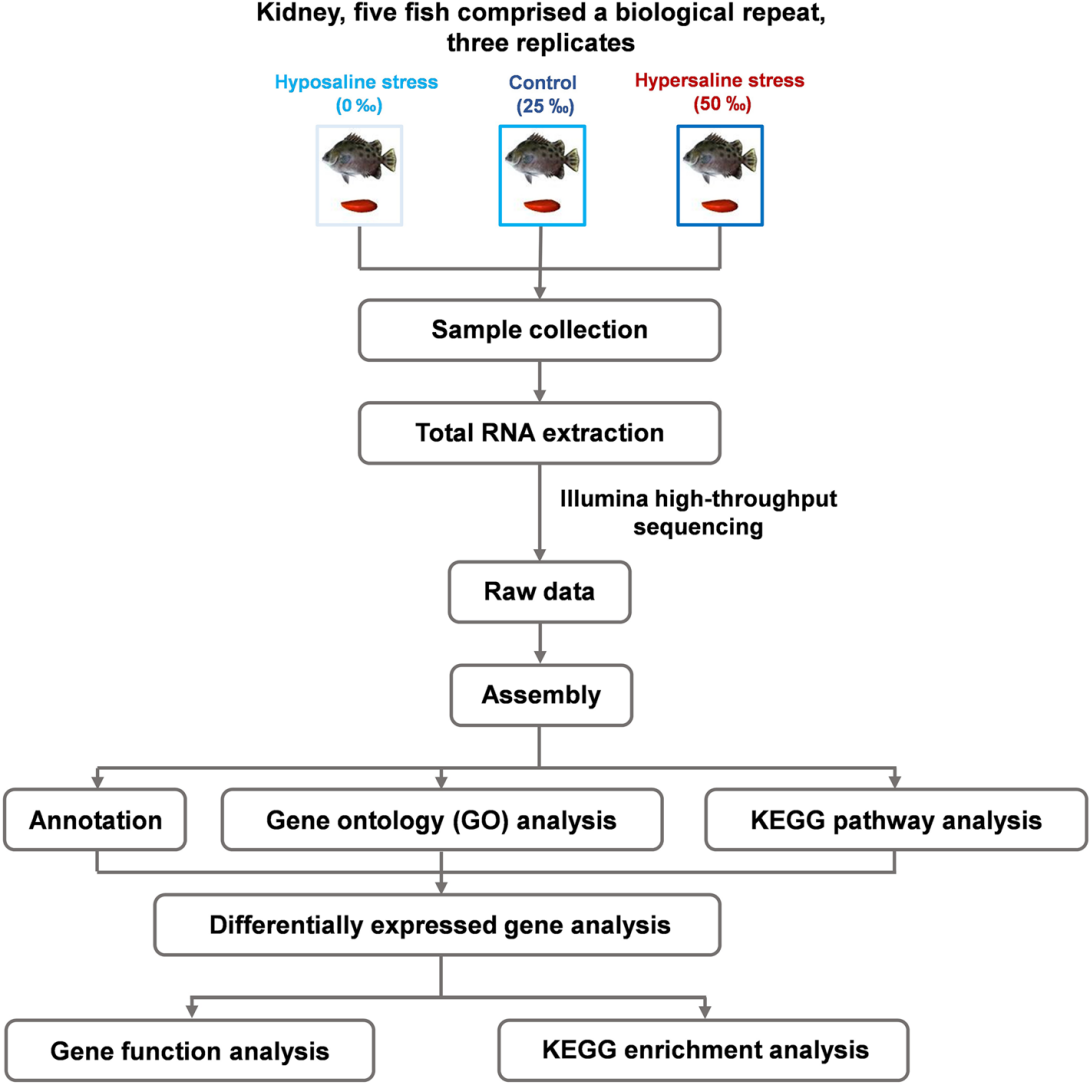
General transcriptomic analysis workflow used in the present study. 0‰ = 0 mOsm/L, 25‰ = 670 mOsm/L, and 50‰ = 1420 mOsm/L. The image of *S. argus* was taken by ourselves

The filtered transcriptome generated via de novo assembly was used as a reference transcriptome for RNA-Seq expression analysis. Raw reads generated from each sample were mapped to the reference transcriptome, and the relative expression level of each unigene was determined using FPKM values calculated using RSEM software [32]. The resulting data matrix, which contained the expression values (FPKM) for the samples from all three salinity acclimation groups, was generated by the “rsem-generate-data-matrix” script. This data matrix was imported into edgeR 2.14 [38] to identify differentially expressed genes (DEGs) with a false discovery rate (FDR) of *p* < 0.001. The FPKM values of the DEGs were normalized by log2 transformation and median centered. Hierarchical cluster analysis was performed based on Euclidean distance using a Trinity Perl script [30]. Genes with an adjusted *p*-value < 0.001 and log_2_|fold-change| ≥ 1 were considered significant. GO and KEGG enrichment analysis were performed to identify the biological functions of DEGs. The transcriptomic analysis workflow used in the present study is shown in Fig. 1.

### cDNA preparation and RT-qPCR validation

The total RNA of each sample was extracted using TRIzol Reagent (Invitrogen, USA). RNA integrity was determined using agarose gel electrophoresis and quantified using a Nanodrop-2000 spectrophotometer (Thermo Scientific, USA). Only RNA samples with an A_260_/A_280_ ratio in the range of 1.8 to 2.0 were employed. Complimentary DNA (cDNA) was synthesized via the reverse transcription (RT) of 1 μg of total RNA with oligo (dT) primers using a PrimeScript RT Reagent Kit with gDNA Eraser (Takara, Japan) and then stored at − 20 °C.

Subsequently, RNA samples were re-analyzed using quantitative PCR (qPCR) to validate the RNA-Seq results. The analysis focused on genes related to dopamine transport. Primer 3 (http://bioinfo.ut.ee/primer3-0.4.0/primer3/input.htm) was used to design gene-specific primers according to the assembled transcriptome sequences. The *β-actin* gene was used as an internal control to normalize the expression value of the target transcript based on the variation in cDNA abundance, as reported previously [39]. Reactions were performed in triplicate for each sample using SYBR Premix Ex Taq II (Takara, Japan) and an ABI Prism 7500 sequence analysis system (Applied Biosystems, USA). The fluorescence intensities of the gene products of the control and treatment groups, as measured by cycle threshold (Ct) values, were compared and converted to fold-differences by relative quantification using the Relative Expression Software Tool 384 v.1 (REST) [40]. The reaction mixture contained 5 μL of SYBR Premix Ex TaqTM II (2×), 0.2 μL of ROX Reference Dye II (50×), 3.4 μL of ddH_2_O, 0.2 μL of forward and reverse primers (10 μM each) and 1 μL of cDNA. The reaction was performed under the following conditions: 34 s at 95 °C; 40 cycles of 5 s at 95 °C and 30 s at 60 °C; 15 s at 95 °C; 1 min at 60 °C; and 15 s at 95 °C (final dissociation). The data were collected at 60 °C. The RT-qPCR primer sequences used in this study are listed in Table 1. To verify the primer specificities, the amplified products of all primers were sequenced (Sangon, China).

**Table 1:** Primers used in RT-qPCR

Gene	Primer sequence (5′-3′)	Amplicon size (bp)
*CaMKIIα*	F: TCCCTGAACCTCTATGGCGA	111
	R: ATGGGGGTGGTACACAGAGA	
*dnm*	F: CACAAAGCGGGGAGAGGTAA	149
	R: CTGGCTTTTGTGGCAGGTTG	
*pkcβ*	F: TCGCACTGTGGTTCACATCA	175
	R: GCAAGCAAGCAGCAGAGATG	
*akt1*	F: GCAGCACAGCATCAAGAGAG	207
	R: TGCCAGACATGCAAAGAATGC	
*dat*	F: GCTTATTGAAGCCATCGGCA	138
	R: AACAGGAGGAAGCAGGGACT	
*lrrk2*	F: AACCTCTGGTGCTCGGAAAC	171
	R: TACAGACTACGGCATCGCTC	
*ace*	F: CATCCACTATGCTCCCTGCC	168
	R: CGCAGCAATATTTCCAGCCC	
*apn*	F: TTGGACAGGGCTATGGTTCC	82
	R: TCCCCTGTTTTGATGAGCCA	
*pink1*	F: CGGCGAACATTTCCCTCTGT	193
	R: TCCACAGAAAAGCGGAGCAC	
*nka α1*	F: AGCTGAAAGACATGACCGCA	183
	R: TGTCAGCCTTCTTCAGAGCG	
*β*-*actin*	F: CTGTGCTGTCCCTGTATG	151
	R: TAGTCTGTGAGGTCACGG	

(*CaMKIIα* calcium/calmodulin-dependent protein kinase II alpha, *dnm* dynamin, *pkcβ* protein kinase C beta, *akt1* RAC-alpha serine/threonine-protein kinase, *dat* dopamine transporter, *lrrk2* leucine-rich repeat kinase 2, *ace* angiotensin-converting enzyme, *apn* aminopeptidase N, *pink1* PTEN-induced putative kinase 1, *nkaα1* Na^+^/K^+^-ATPase subunit alpha 1, *F* forward, *R* reverse)

Each reaction had a single peak in the melt curve that corresponded to a single product. The amplification efficiencies of both the target and reference genes ranged from 99.6 to 100.3%. The relative transcript levels of different genes were determined by subtracting the cycle threshold (Ct) of *β-actin*, which was utilized as a calibrator or internal control, using the following formula: ΔCt = Ct (sample) - Ct (calibrator). All data were analyzed by the 2^-ΔΔCt^ method as described in Mu et al. [9]. Relative transcript expression values were presented as a fold-change relative to the expression value of the control group (25‰).

### Primary kidney cell culture

Kidney tissue was obtained for primary cell culture from a healthy 25‰ SW-acclimated *S. argus* fish as described in Lakra et al. [41]. In brief, harvested kidney tissue was saturated with L-15 cell culture medium containing antibiotics (1000 U penicillin and 1000 U streptomycin). Subsequently, the kidney tissue was minced thoroughly with scissors and transferred to 25 cm^2^ cell culture flasks containing 8 mL of growth medium with 20% fetal bovine serum (FBS). The primary cells were incubated at 28 °C, and one-half of the medium was changed every 3 days for 2 weeks. When the migrating cells formed a complete monolayer, the cells were trypsinized with a 0.25%-trypsin solution and transferred into a fresh T25 flask with fresh medium containing only 10% FBS. The cell cultures were then maintained at 28 °C.

For osmotic stress experiments, primary cells were subjected to hypoosmotic (100 mOsmol/L) and hyperosmotic (600 mOsmol/L) media, whereas control cultures were exposed to fresh isosmotic medium (300 mOsmol/L). The culture media and cells were collected 24 h after salinity challenge.

### Determination of dopamine concentration

The dopamine concentrations in serum and culture media were measured using the Dopamine ELISA Kit specific for fish (Cusabio, China, https://www.cusabio.com/) according to the manufacturer’s instructions. The 96-well microtiter plate provided in this kit was pre-coated with anti-dopamine monoclonal antibody, and no signification cross-reactivity or interference between dopamine and its analogs was observed. The precision of the assay was determined by the repeated measurement of control or experiment samples. Both positive and negative controls (phosphate buffer solution (PBS)-only and blank, respectively) were analyzed in this assay. After all reactions, the optical density of each well was measured at 450 nm within 10 min of reaction termination using a Synergy H4 Hybrid Multi-Mode Microplate Reader (BioTek, USA). The dopamine concentration of each sample was determined according to a standard curve.

### Immunofluorescence and Western blot analysis of NKAα1

The kidney tissue from a healthy 25‰ SW-acclimated *S. argus* fish was harvested to prepare kidney slices. The localization of NKAα1 was studied using immunofluorescence staining and was performed as follows. Briefly, sections were incubated in 5% skim milk for 1 h at 37 °C, incubated with primary antibody (1:400 dilution) in PBS containing 0.5% Triton X-100 (PBST) overnight at 4 °C, incubated with fluorescein isothiocyanate- (FITC-) conjugated secondary antibody (Abcam, UK) (1:1000 dilution) in PBST for 3–4 h at room temperature. Nuclei were stained with 4′,6-diamidino-2-phenylindole (DAPI, 300 nM, Sigma-Aldrich, USA) in PBS for 5 min. Slices were visualized with a LSM510 confocal laser scanning microscope (Zeiss, Germany). Negative controls were incubated with normal rabbit serum.

The total protein of each kidney sample was extracted using a Protein Extraction Kit (Invent, USA), and the protein concentration was then determined using a BCA Protein Assay Kit (Merck, Germany). Total protein (75 μg) was separated on 12% sodium dodecyl sulfate- (SDS-) polyacrylamide gels and transferred to polyvinylidene fluoride (PVDF) membranes. Following blocking in a 5%-skim milk/PBS solution (Bio-Rad, USA), membranes were incubated with anti- Na^+^/K^+^ ATPase alpha 1 antibody (Abcam, UK) (1:5000 dilution) in PBS overnight at 4 °C. Membranes were washed and incubated with horseradish peroxidase- (HRP-) conjugated secondary antibody (Abcam, UK) (1:5000 dilution) for 2 h at room temperature. Chemiluminescence was used to quantify secondary antibody conjugation using a Universal Hood II gel documentation system (Bio-Rad, USA).

### Measurement of NKA activity

Total protein from primary renal cells and kidney tissues was isolated and quantified using a BCA Protein Assay Kit (Merck, Germany). Subsequently, NKA activity was measured using a Sodium-Potassium-ATPase Kit (Jiancheng, China) according to the manufacturer’s instructions. Activity was expressed in μmolPi/mgprot/h.

### Determination of Na^+^ and K^+^ content in culture media via ion chromatography

Culture medium samples were diluted with deionized water (hypotonic medium, 1:60 dilution; isotonic medium, 1:180 dilution; hypertonic medium, 1:280 dilution) and filtered with Nalgene syringe filters (0.22 μm, Thermo Scientific, USA). Analyses were conducted using a Dionex™ ICS-1500 ion chromatography system (Thermo Scientific, USA) with an AS-DV automated sampler.

### Statistical analyses

All data were analyzed for normality (Kolmogorov-Smirnov’s test) and homoscedasticity of variance (Levene’s test). Statistically significant differences between the treatment and control groups were determined using one-way analysis of variance (ANOVA), followed by Student’s *t*-test with SigmaStat software (Systat Software, USA). *p* < 0.05 was considered statistically significant (* *p* < 0.05, ** *p* < 0.01, *** *p* < 0.001).

## Results

### De novo assembly and functional annotation

Total RNA was isolated from an *S. argus* kidney subjected to different salinity acclimation conditions to produce a large-scale *S. argus* transcriptome. A total of 1,023,047,770 paired-end reads (150 bp each) were obtained. After removing low-quality reads, 575,312,926 clean reads remained and were used for de novo transcriptome assembly, resulting in 186,397 unigenes (> 250 bp). By selecting transcripts with FPKM ≥ 1, 43,933 unigenes were determined as viable for downstream analysis. The N50 value of these unigenes was 3118 bp, with an average length of 2022 bp (Table 2). The length distribution of these unigenes is shown in Additional file 1: Figure S1. The high quality of sequence reads and assembly was the foundation for all subsequent analyses. All generated sequence reads were deposited in the NCBI SRA database with the BioProject accession: PRJNA362802.

**Table 2:** Summary statistics of *S. argus* transcriptome. *FPKM: fragments per kilobase per transcript per million mapped reads

Sequencing	Number	
Before trimming		
Raw reads (pair-end)	1,023,047,770	
After trimming		
Clean reads (pair-end)	575,312,926	
Total nucleotide (bp)	7.22 × 10^10^	
After assembly		
	De Novo assembled	Filtered (FPKM* ≥ 1)
Transcripts	186,397	43,933
N50 length (bp)	2521	3118
N90 length (bp)	462	978
Reads Mapped	1.94 × 10^8^	1.26 × 10^8^
GC content (%)	43.20	45.02
Min length (bp)	224	
Max length (bp)	23,112	
Average length (bp)	1272	2022
Annotation		
NCBI-NR	14,259	
SWISS-PROT	8360	
GO	12,247	
KEGG	11,354	
KOG	3168	

A total of 14,259 (32.5% of the total transcripts) and 8360 unigenes were functionally annotated by NCBI-BLAST searches against protein databases (NR and SWISS-PROT), respectively (Table 2). The top five species for which information and unknown unigenes aligned were all fish and included *Larimichthys crocea* (47.50%), *Stegastes partitus* (16.45%), *Notothenia coriiceps* (7.51%), *Oreochromis niloticus* (5.74%) and *Oncorhynchus mykiss* (5.43%) (Additional file 2: Figure S2). The sequences of assembled unigenes were also subjected to BLASTP searches against the KOG, GO and KEGG databases. The GO and KOG databases are primarily used to describe the functional classification of genes [42, 43]. To obtain a first assessment of the physiological processes occurring in the kidney during salinity stress, GO analysis and KOG functional classification were performed. A total of 12,247 unigenes were assigned to 67 sub-categories of GO terms belonging to the following three categories: ‘biological process (BP)’, ‘cellular component (CC)’ and ‘molecular function (MF)’ (Additional file 3: Figure S3). The most interesting sub-categories were related to ‘hormone secretion’ and ‘channel regulator activity’. For KOG annotation, 12,083 unigenes mapped to the KOG database were clustered into 25 categories, which included the two primary categories of ‘Intracellular trafficking, secretion, and vesicular transport’ (813) and ‘Inorganic ion transport and metabolism’ (763) (Additional file 4: Figure S4). KEGG is a database resource for understanding high-level functions and utilities of genes in biological systems, particularly from large-scale datasets generated by RNA-Seq [44]. To further elucidate the biological pathways involved in the reaction to salinity stress in the *S. argus* kidney, the unigene sequences were mapped using KEGG pathway tools. These results were used to classify 11,354 unigenes into 332 specific pathways (Table 2). Similar to the GO analysis and KOG classification results, ‘organismal systems’ was the most important classification in the KEGG analysis, which contained the sub-classification categories of ‘endocrine system’, ‘nervous system’ and ‘excretory system’. These annotations may provide a valuable resource for the further understanding of specific biological functions in the kidney of *S. argus* under salinity stress.

### Analysis of DEGs for salinity tolerance in *S. argus*

**Fig. 2:**
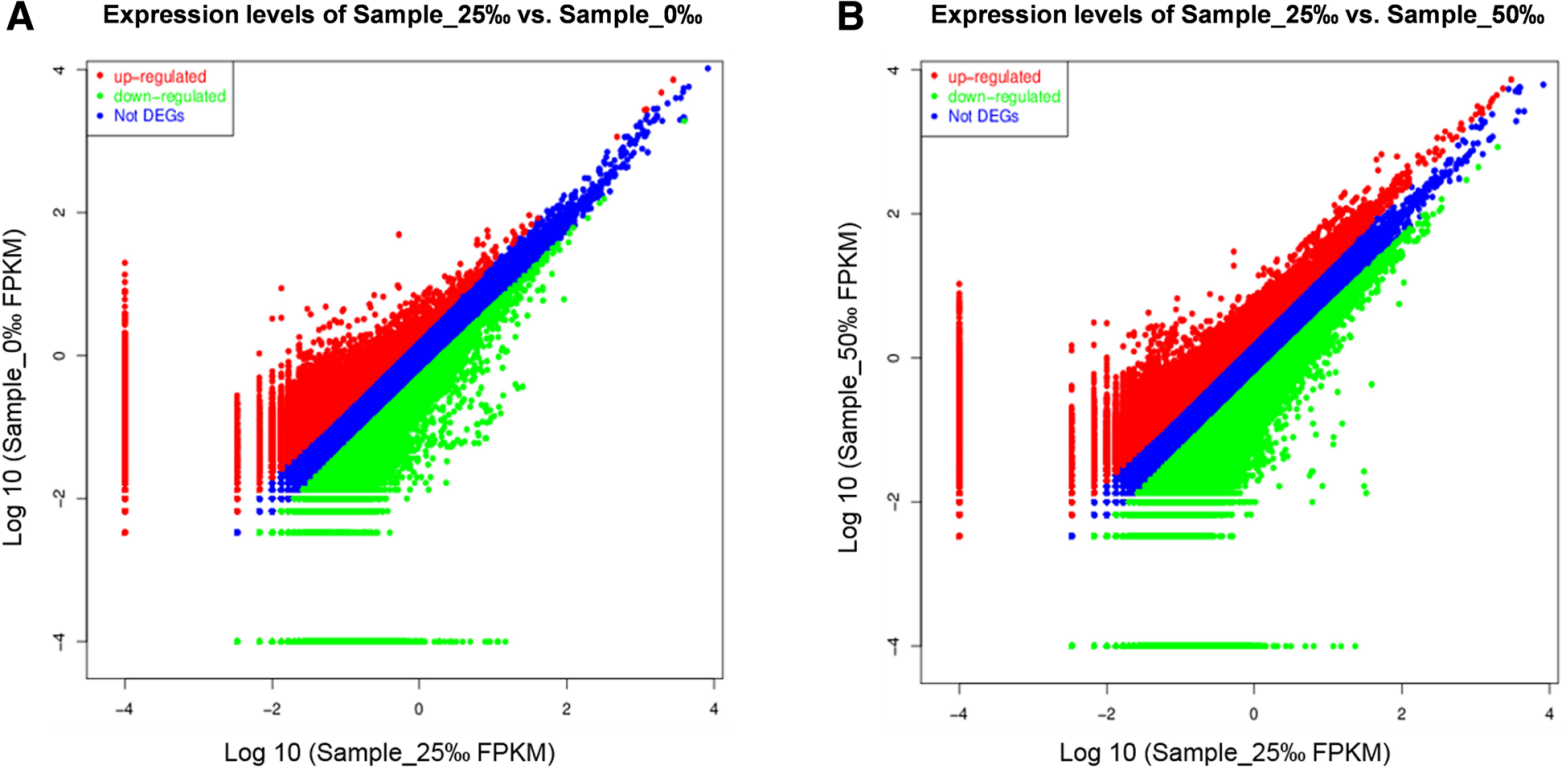
Identification of differentially expressed unigenes displayed by FPKM in (A) Sample_0‰ vs. Sample_25‰ and (B) Sample_50‰ vs. Sample_25‰ using a log_10_ scale. The x-axis (Sample_25‰) and y-axis (Sample_0‰ and Sample_50‰) show the logarithmic value of the normalized expression level of each gene in FPKM (fragments per kb per million fragments)

In comparison with the control group, 19,012 DEGs (9956 up-regulated and 9056 down-regulated) were identified in the FW group. Of those DEGs, 1439 DEGs were significantly expressed in the kidney (*p* < 0.001 and log_2_|fold-change| ≥ 1) (Fig. 2). Similarly, 36,253 DEGs were identified after comparing the control and HW groups: 7413 and 28,840 of those DEGs were up-regulated and down-regulated, respectively. In addition, 2367 DEGs were shown to be significantly expressed upon comparison of the control and HW groups (*p* < 0.001 and log_2_|fold-change| ≥ 1). The results showed that the number of DEGs in the HW group was greater than that in the FW group. This finding suggests hypersaline stress causes greater changes in renal gene expression than FW stress, indicating elevated environmental salinity might have a significant impact on renal function in *S. argus*.

**Fig. 3:**
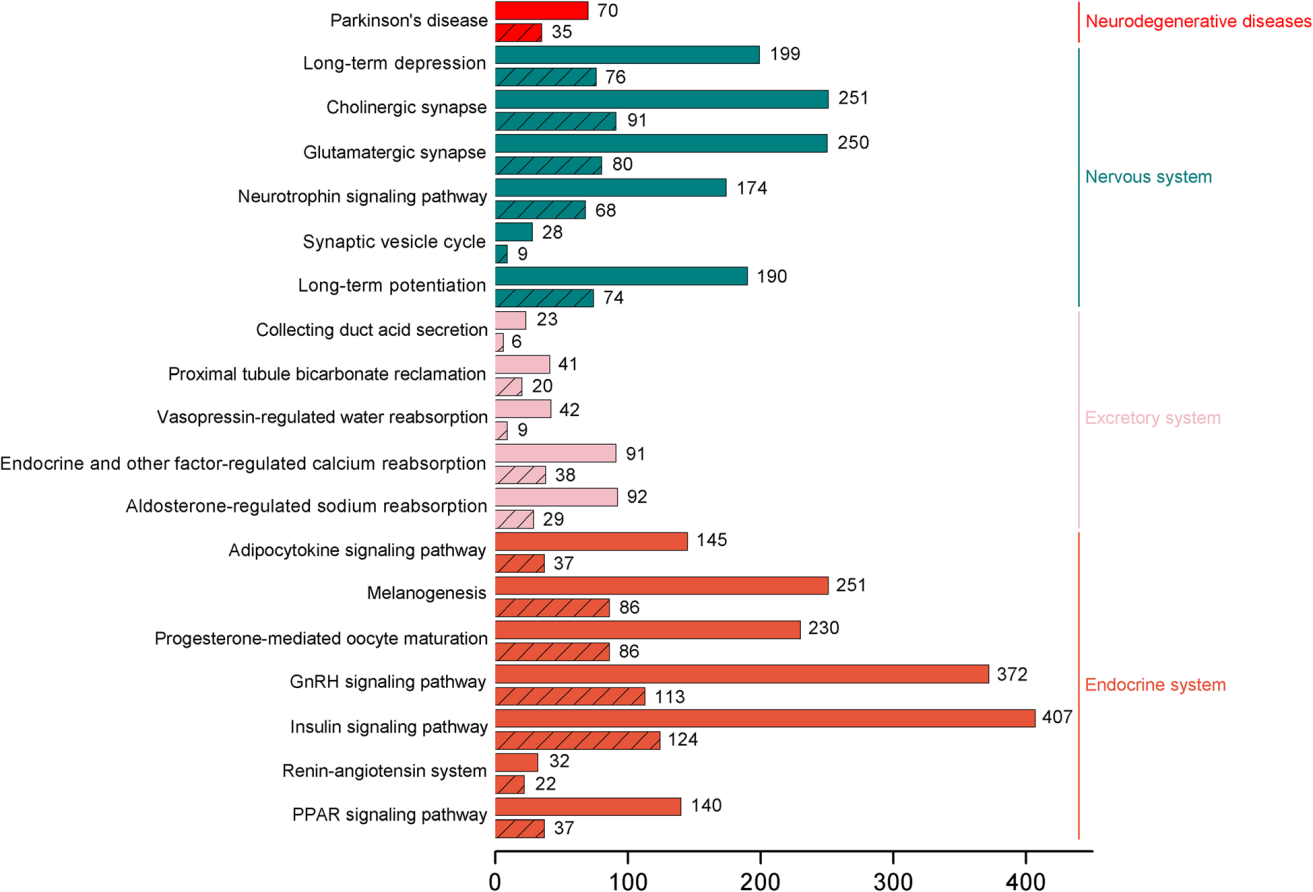
KEGG pathway classification for DEGs. Shaded bars represent KEGG pathway classification for DEGs upon comparison of the FW group (0‰) and control group (25‰). Non-shaded bars represent KEGG pathway classification for DEGs identified after comparison of the HW group (50‰) and control group (25‰)

To explore the potential functions of DEGs in the kidney, all DEGs were assigned to GO and KEGG databases. The significantly differently expressed GO terms (*p* < 0.001) in the DEGs included the following: sodium/chloride symporter activity (GO:0015378), ion channel activity (GO:0005216), neurotransmitter secretion (GO:0007269), synaptic transmission involved in micturition (GO:0060084), regulation of ion transmembrane transport (GO:0034765) and synaptic vesicle (GO:0008021). GO enrichment analysis indicated hormone secretion and ion transport in the *S. argus* kidney were significantly influenced by exposure to fluctuating salinity levels. In various organisms, hormones are used to communicate between organs and tissues for physiological regulation, including regulation of ion balance. Dopamine can inhibit renal Na^+^ transporters, channels, and pumps, resulting in a decrease in tubular sodium reabsorption and eventually an increase in sodium excretion. The results of this study identified the GO terms related to the dopamine system (dopamine receptor activity coupled via Gi/Go, GO:0001591; response to amphetamine, GO:0001975) (*p* < 0.001). Similar to the GO annotation results, ‘endocrine system’ is also the primary classification in the KEGG analysis, which contained ‘renin-angiotensin system’. For ‘nervous system’ and ‘excretory system’, the ‘synaptic vesicle cycle’ and ‘aldosterone-regulated sodium reabsorption’ pathways were identified. Interestingly, the KEGG pathway enrichment analysis showed the ‘Parkinson’s disease’ pathway related to dopamine dysfunction was significantly enriched for DEGs (Fig. 3). These results revealed the renal dopamine system might play an important role in osmoregulation.

### RT-qPCR validation of candidate genes associated with dopamine transport

**Fig. 4:**
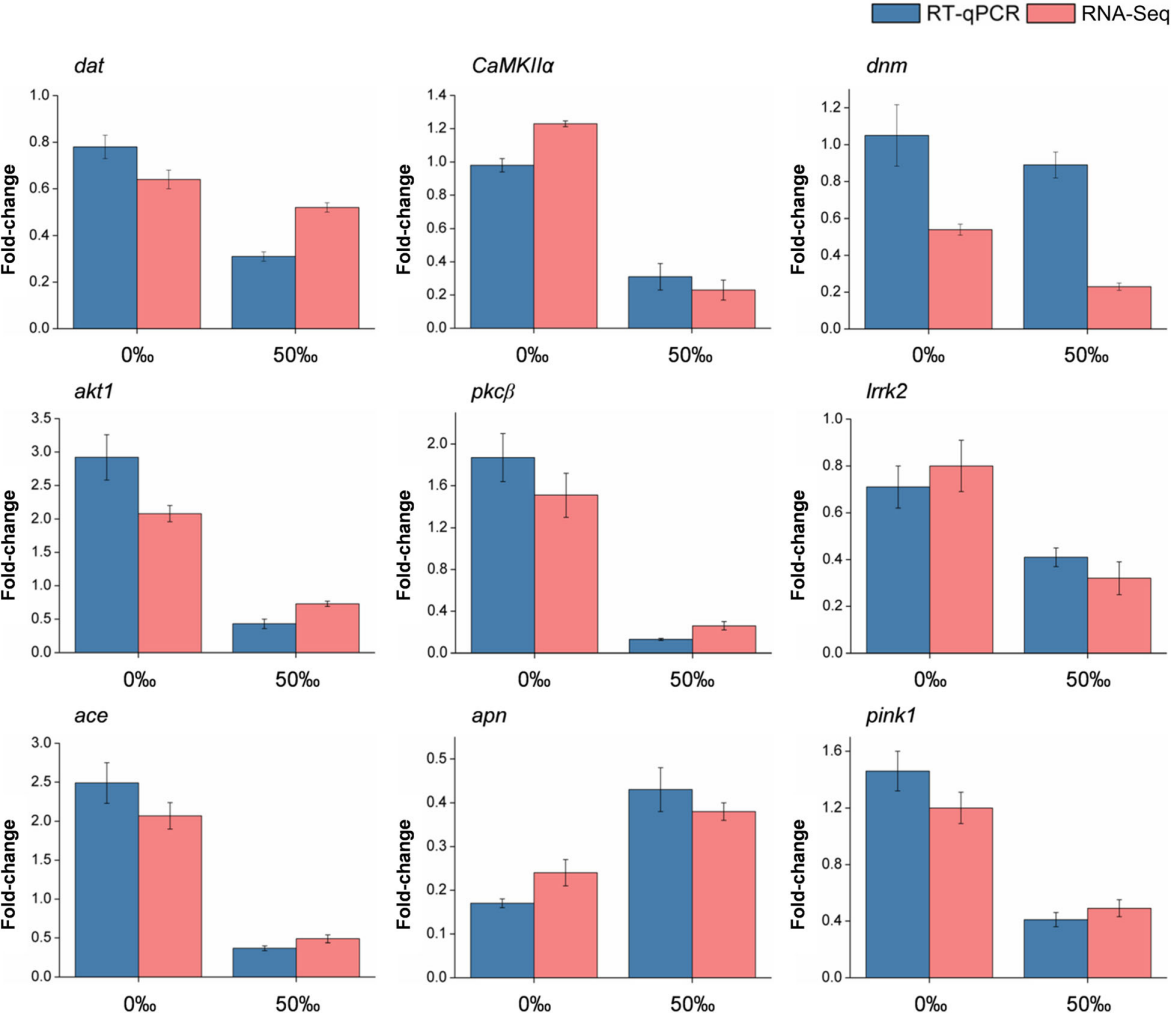
Expression profiles of the nine DEGs associated with dopamine transport based on RNA-Seq (pink) and RT-qPCR (blue) analyses of changes in transcript expression following salinity acclimation. *β-actin* served as a reference gene. The results are presented as the mean ± standard error of the mean (SEM) with three replicates

Dopamine signaling was dependent on the extracellular dopamine level, which was regulated by a dopamine release/reuptake mechanism. According to the functional annotation results, 10 genes involved in dopamine release/reuptake were selected from the DEGs with a log_2_|fold-change| ≥ 1 for RT-qPCR analysis (Fig. 4).

The RT-qPCR results showed RAC-alpha serine/threonine-protein kinase (*akt1*), an inhibitor of dopamine transporter (DAT) internalization, was significantly up-regulated in the kidney after FW acclimation. In addition, a significant increase in angiotensin-converting enzyme (*ace*), which promotes dopamine release, was observed. These results suggest long-term hypoosmotic stress inhibits DAT internalization, which can directly promote dopamine reuptake, although ACE-induced dopamine release may be enhanced.

In the HW fish kidney, expression of the genes involved in dopamine reuptake (*dat*), DAT phosphorylation (calcium/calmodulin-dependent protein kinase II alpha, *CaMKIIα*; protein kinase C beta, *pkcβ*), DAT internalization (*akt1*) and vesicular traffic-mediated dopamine release (PTEN-induced putative kinase 1, *pink1*; leucine-rich repeat kinase 2, *lrrk2*; *ace*; aminopeptidase N, *apn*) was all significantly reduced. These results indicate phosphorylated DAT- and vesicular traffic-mediated dopamine release was weakened in the kidney of *S. argus* during chronic hypersaline stress, and the decreased expression of DAT fundamentally reduced the influx of extracellular dopamine.

### Dopamine content in vivo and in vitro

**Fig. 5:**
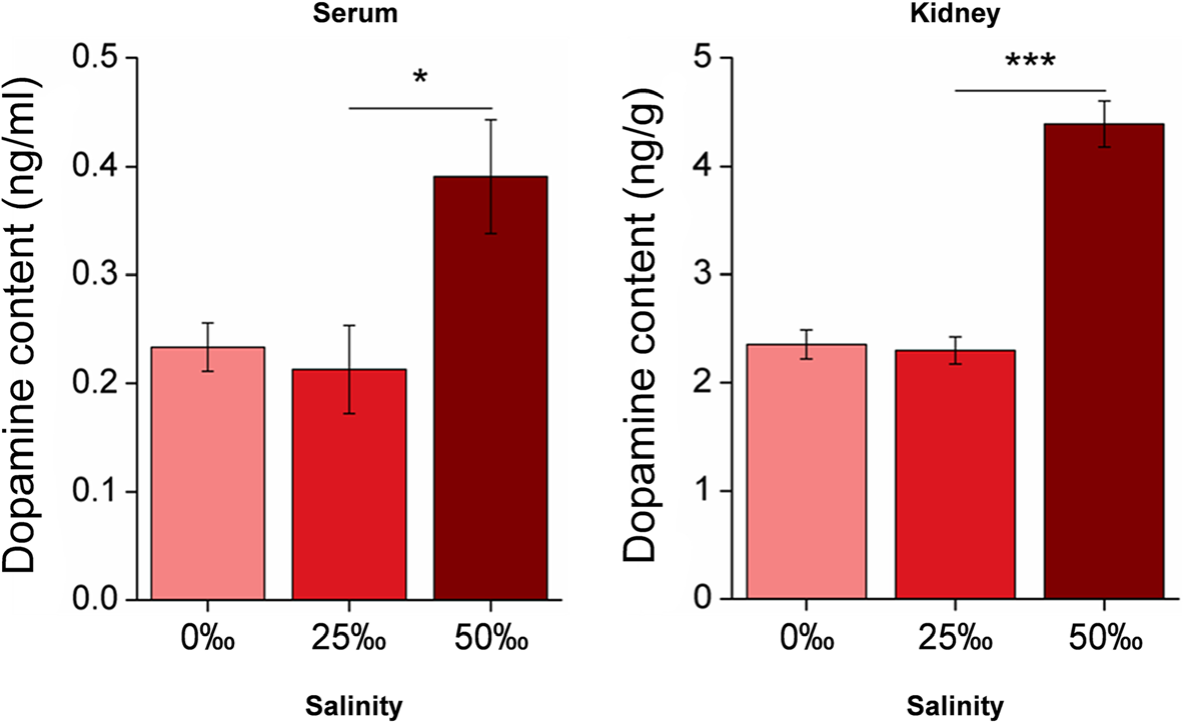
Changes in the dopamine concentration present in serum and kidney samples from *S. argus* fish acclimated to different salinities. Dopamine concentrations in the serum and kidney of 25‰ SW-reared *S. argus* were used as controls. *p < 0.05; ***p < 0.001

Changes in the expression of the genes mentioned in the previous section ultimately affect the secretion of dopamine. Therefore, the concentrations of dopamine in vivo and in vitro under different salinities were measured. During chronic acclimation to FW, dopamine concentrations in both serum and kidney tissues of *S. argus* remained relatively stable. Compared with the control group, significant increases (*p < 0.05, ***p < 0.001) in the dopamine concentration were observed in the serum (from 0.21 ± 0.04 ng/ml to 0.39 ± 0.05 ng/ml) and kidney tissues (from 2.30 ± 0.13 ng/g to 4.39 ± 0.21 ng/g) of HW fish (Fig. 5).

**Fig. 6:**
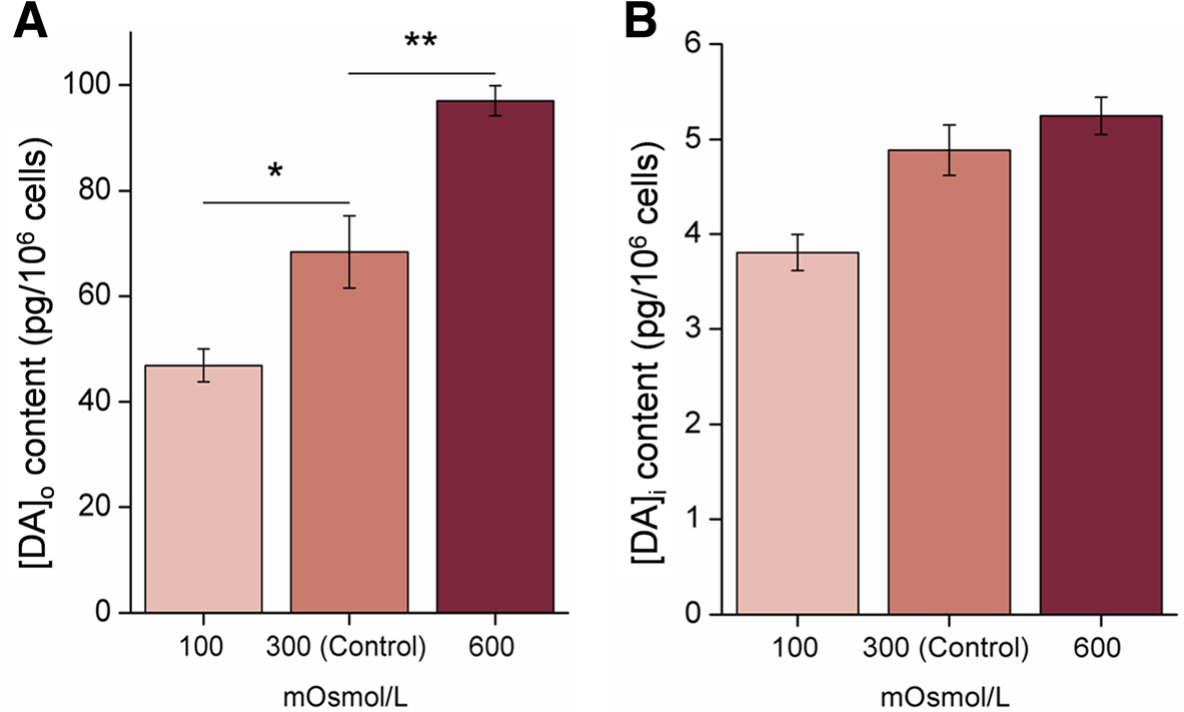
Effect of salinity stress on dopamine transport in vitro. [DA]_o_ represents the extracellular DA concentration, and [DA]_i_ represents the intracellular DA concentration. *p < 0.05; **p < 0.01

The in vitro dopamine concentration in the culture medium ([DA]_o_) of primary renal cells exposed to hypoosmotic shock (100 mOsmol/L) decreased significantly (from 68.37 ± 6.87 pg/10^6^ cells to 46.90 ± 3.09 pg/10^6^ cells) at 24 h post-treatment (*p < 0.05) but increased in the hypertonic medium (600 mOsmol/L) (from 68.37 ± 6.87 pg/10^6^ cells to 97.01 ± 2.84 pg/10^6^ cells) (**p < 0.01) (Fig. 6) compared with cells pre-treatment. No significant differences were observed in the intracellular dopamine level ([DA]_i_) when compared with cells pre-treatment.

### NKA expression and activity

**Fig. 7:**
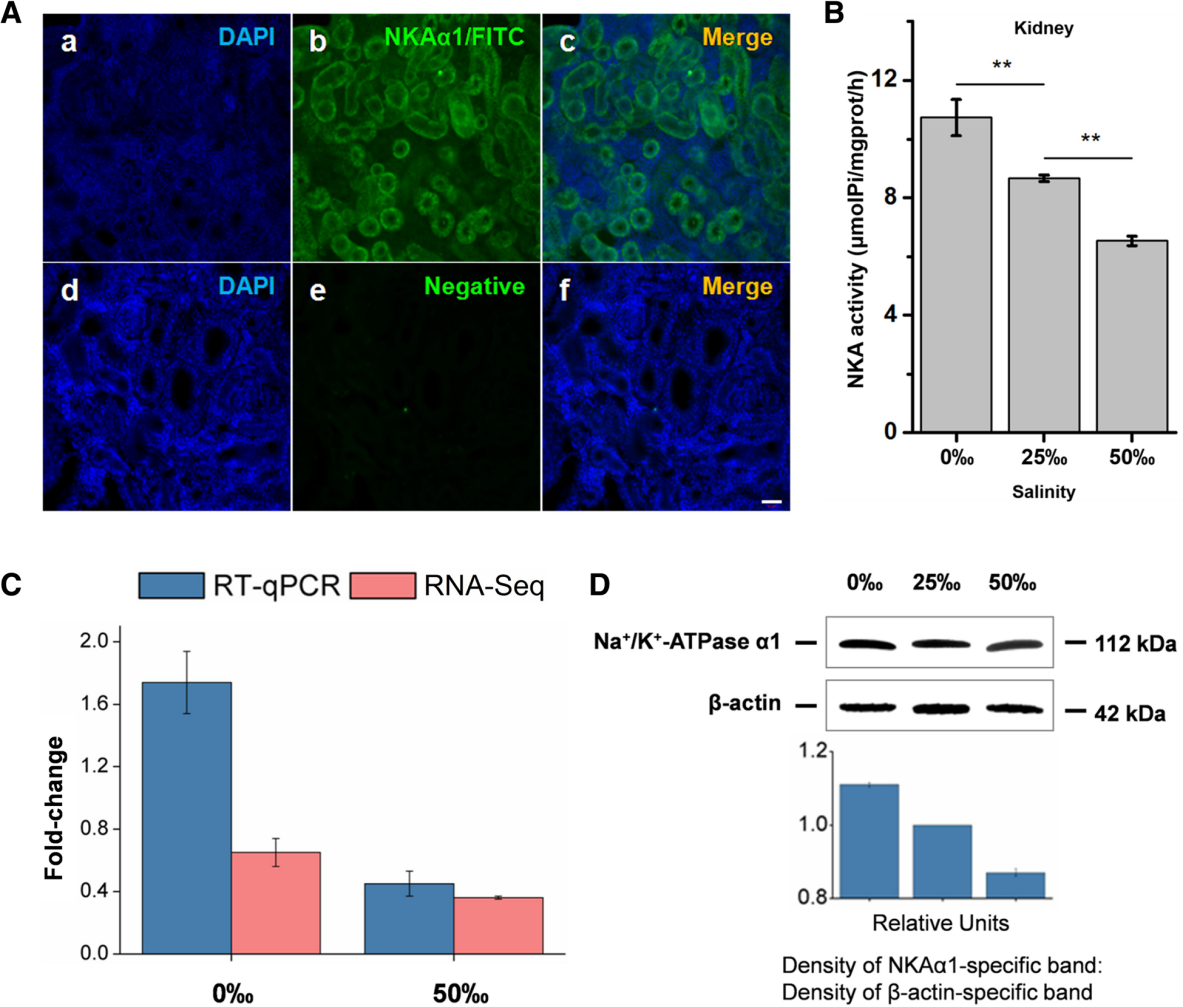
Changes in NKAα1 expression and NKA activity in *S. argus* kidneys during salinity tolerance experiments. **a** Immunolocalization of NKAα1 in *S. argus* kidney. NKAα1 was visualized using a primary antibody against NKAα1 (green, b), and nuclei were identified by staining with DAPI (blue, a and d). Negative control was incubated with normal rabbit serum (e). Scale bar = 25 μm. **b** NKA activity in kidneys isolated from fish acclimated to different salinities. **c** Expression profile of *nkaα1* from RNA-Seq (pink) and RT-qPCR (blue) analyses of changes in transcript expression following salinity acclimation. *β-actin* served as a reference gene. The results are presented as the mean ± SEM with three replicates. **d** Western blot analysis of NKAα1 and *β-actin* in *S. argus* kidney during salinity stress

Immunofluorescence staining was conducted to determine the localization of NKAα1 in the SW-acclimated *S. argus* kidney. Renal sections were double-stained with primary antibody against NKAα1 (green, Fig. 7a-b) and DAPI (blue, Fig. 7a-a and Fig. 7a-d). Negative control incubated with normal rabbit serum was showed in Fig. 7a-e. The results showed NKAα1 was widely expressed in renal tubules.

Chronic exposure to FW (0‰) slightly altered the mRNA and protein expression of NKAα1 and significantly increased NKA activity (from 8.61 ± 0.20 μmolPi/mgprot/h to 10.84 ± 0.79 μmolPi/mgprot/h) (**p < 0.01). In the HW group kidneys, both NKAα1 expression and NKA activity (6.53 ± 0.37 μmolPi/mgprot/h) were significantly inhibited (**p < 0.01) (Fig. 7b, c and d).

### Analysis of Na^+^ and K^+^ transport in vitro

**Fig. 8:**
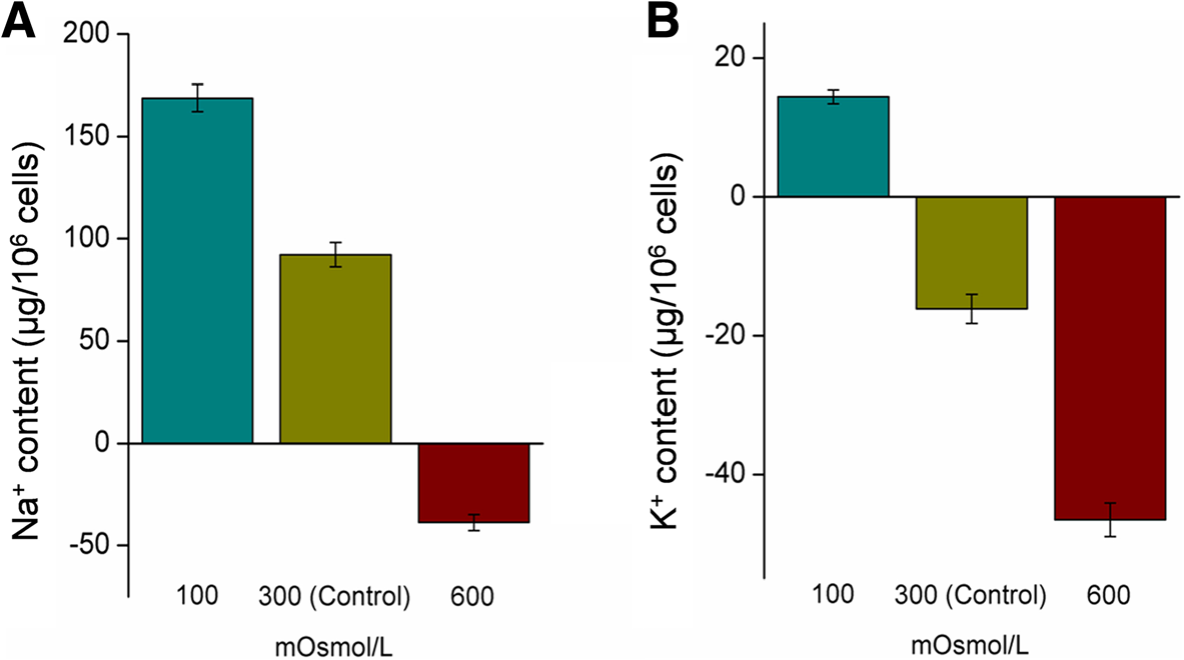
Analysis of Na^+^ and K^+^ transport in renal primary cells under different osmotic stress conditions using an ion chromatography system

The concentrations of Na^+^ and K^+^ in culture media at different osmotic pressures were determined using ion chromatography. Figure 8 presents the differences between the actual and initial ion concentrations in the culture media: a difference greater than 0 indicates efflux (out of the cell), and a difference less than 0 indicates influx (into the cell). After exposure of renal primary cells to the hypotonic medium, the Na^+^ efflux was almost twice (166.14 ± 4.39 μg/10^6^ cells) that outflowed under isosmotic conditions (86.22 ± 4.23 μg/10^6^ cells). In addition, K^+^ was pumped out of cells (14.46 ± 1.94 μg/10^6^ cells) during chronic hypotonic stress, although an influx of K^+^ was observed under isosmotic conditions (16.17 ± 2.91 μg/10^6^ cells). In contrast, exposure of renal primary cells to hypertonic medium promoted Na^+^ (35.50 ± 1.04 μg/10^6^ cells) and K^+^ (46.59 ± 5.24 μg/10^6^ cells) influx to maintain the homeostasis of kidney cells.

## Discussion

Salinity adaptation in euryhaline teleosts is a complex process involving physiological responses in several osmoregulatory organs. In fish, the kidney is an important osmoregulatory organ involved in maintaining osmotic homeostasis [13, 45]. Functional changes are reflected by the differential expression of various genes in the kidney. Most studies on osmoregulation in euryhaline teleosts have not provided comprehensive information on its complex functional mechanisms. For producing a comprehensive reference transcriptome, all raw reads from the groups exposed to three different salinity levels were pooled (more than 1 billion paired-end reads) and assembled in the present study. A high-quality reference transcriptome was generated from the raw reads by applying the Trinity assembler, which is based on a de Bruijn graph algorithm for short-read transcript assembly. In total, 2367 significant DEGs were identified in the kidneys of *S. argus* harvested from the HW and FW groups.

Similar to other vertebrates, the regulation of salinity adaptation in teleosts is dependent on the endocrine system, which mediates many physiological processes to maintain a salt and water balance [46, 47]. Dopamine, a catecholamine hormone, directly affects the expression and activity of Na^+^ transporters via its specific receptors [19]. In this study, GO functional annotation analysis revealed a relatively large number of transcripts involved in dopamine receptor-mediated signaling transduction, and KEGG analysis revealed an enriched pathway related to dopamine transport. Interestingly, the transcriptional data from this study indicated no significant changes occurred in the expression of dopamine receptors (log_2_|fold-change| < 1). Thus, the dopamine availability at dopamine receptors and the timing and magnitude of dopamine signaling were dependent on the regulation of extracellular dopamine levels. This finding was consistent with the assumption that chronic salinity tolerance has a considerable influence on dopamine signaling by interfering with its transport, which directly affects the dopamine-mediated regulation of the Na^+^ balance.

ELISA assay results showed the dopamine levels in serum and the kidney tissues of *S. argus* increased significantly during hypersaline tolerance but remained relatively stable under hyposaline conditions (Fig. 5). In addition to the kidney, non-neuronal dopamine was synthesized and secreted in the pancreas [20]. The interference of multiple sources of non-neuronal dopamine, could be avoided in in vitro experiments, and the concentrations of extracellular and intracellular dopamine were determined in the present study. A significant increase (*p* < 0.01) of extracellular dopamine was observed after the exposure to hyperosmotic medium, but its concentration decreased significantly (*p* < 0.05) in hypoosmotic medium. These results indicated changes of osmotic pressure in the culture medium greatly influenced extracellular dopamine levels. To avoid cytotoxicity, intracellular dopamine levels remained relatively stable regardless of whether cells were exposed to hypoosmotic or hyperosmotic stress. Apart from regulating the expression and activity of Na^+^ channels via dopamine receptors, dopamine can also promote natriuresis via increasing renal blood flow and the glomerular filtration rate in vivo [48]. This shed light on the phenomenon that dopamine remained stable in vivo but decreased significantly in vitro under hypoosmotic stress in this study.

Two major regulators of extracellular dopamine are vesicular traffic-mediated release and DAT-mediated reuptake [49]. The primary mechanism for the clearance of extracellular dopamine is reuptake mediated by DATs, which is governed by the number of functional DATs in the membrane [50]. This study indicated chronic hypersaline stress induced the reduction of DAT-mediated dopamine clearance by inhibiting DAT expression. Previous studies have shown that reduction of dopamine influx by DATs can increase extracellular dopamine, and thus inhibits the release of intracellular dopamine [51, 52].

In the present study, the expression levels of vesicular traffic-mediated dopamine release-related genes (*pink1*, *lrrk2*, *ace* and *apn*) were significantly reduced (*p*-value < 0.001 and log_2_|fold-change| ≥ 1). LRRK2 is a member of the leucine-rich repeat kinase family. Notably, mutations in *lrrk2* are the most common genetic cause of Parkinson’s disease, a disease in which imbalanced synaptic transmission has been implicated as a causal factor [53–57]. Previous studies have shown LRRK2 regulates synaptic vesicle storage and overexpression of *lrrk2* increases striatal dopamine release [56, 58, 59]. The results of this study revealed the expression level of *lrrk2* was decreased by hypersaline stress. In addition, *pink1*, which is intimately involved with mitochondrial quality control by identifying damaged mitochondria and targeting specific mitochondria for degradation, was inhibited under the hypersaline condition. Mitochondria play a crucial role in synaptic function, consistent with the high energy demand of synaptic terminals, by restoring ionic gradients following synaptic transmission and reloading synaptic vesicles with neurotransmitters [60, 61]. PINK1 deficiency affects synaptic function because the reserve pool of synaptic vesicles is not mobilized during rapid stimulation, which results in a decrease in dopamine efflux [62, 63]. In addition to LRRK2 and PINK1, the renin-angiotensin system (RAS) participates in dopamine efflux mediated by vesicular transport and was initially considered a circulating humoral system that regulated blood pressure and Na^+^/water homeostasis [64]. As an essential enzyme in the RAS system, ACE catalyzes the conversion of angiotensin I (Ang I) to Ang II, which is hypothesized to induce dopaminergic cell death [64, 65]. The contribution of RAS hyperactivation to dopaminergic degeneration can be inhibited by ACE inhibitors [64]. Accordingly, ACE could possess peptidase activity associated with the stimulation of dopamine release [66, 67]. Notably, Ang II must be metabolized to Ang III to increase natriuresis [68]. The inhibition of aminopeptidase N (APN), which is involved in the conversion of locally administered Ang III to Ang IV, causes a dose-dependent decrease in extracellular dopamine abundance [69]. Thus, dopamine release was reduced during hypersaline stress because the expression of several genes (*pink1*, *lrrk2*, *ace* and *apn*) associated with vesicular traffic-mediated dopamine release was inhibited.

In addition to vesicular transport, phosphorylated DATs perform the opposite function to that of DATs. An amino acid sequence analysis of DATs revealed the presence of several consensus sites for protein kinase phosphorylation via protein kinase C (PKC) and calcium/calmodulin-dependent protein kinase [51, 70]. Previous studies indicated surface DAT phosphorylation is increased in the presence of PKC and CaMKII activity, causing the release of intracellular dopamine and stimulation of dopamine signaling [71]. In the present study, decreased *CaMKIIα* and *pkcβ* expression could have produced negative effects on dopamine release mediated by phosphorylated DATs during high-salinity tolerance experiments.

Cell-surface DAT levels are sensitive to changes in the cell membrane potential, which can rapidly drive DAT internalization, which can alter the capacity of DATs to increase extracellular dopamine levels. Known also as protein kinase B, AKT participates in multiple cellular processes and plays an essential role in the homeostatic regulation of DAT activity in mammals [50]. The inhibition of AKT decreases dopamine reuptake capacity and leads to the redistribution of DATs away from the plasma membrane, which decreases dopamine clearance efficiency and provides an additional mechanism by which this DAT substrate increases the extracellular dopamine concentration [72]. In FW fish, the renal *akt1* gene, a member of the AKT subfamily of genes, was significantly up-regulated. This finding suggested hypo-salinity acclimation may promote extracellular dopamine clearance by increasing DAT activity and inhibiting DAT internalization by AKT1. In addition, an increase in the mRNA expression of *ace*-induced dopamine release was observed in FW fish. This phenomenon may be a compensatory mechanism for the increase in extracellular dopamine clearance by DATs.

The physiological functions of DAT in dopamine removal are coupled with the translocation of 2 Na^+^ ions and 1 Cl^−^ ion [49]. Therefore, fluctuations in environmental salinity can directly affect extracellular dopamine clearance by affecting the binding of dopamine to DATs. Furthermore, extracellular and intracellular Na^+^ ions are major determinants of renal dopamine transport [48]. DATs participate in dopamine reuptake using energy provided by the Na^+^ gradient, which is generated by NKA. NKA consists of α-catalytic and β-glycoprotein subunits and plays a crucial role in maintaining osmotic homeostasis [73, 74]. Blocking NKA or promoting a decrease in the external Na^+^ concentration dramatically impairs or completely suppresses DAT-mediated extracellular dopamine clearance [75]. Herein, *nkaα1* was identified from the renal transcript library of *S. argus*. The α-subunit of NKA is a transmembrane protein that cleaves high-energy phosphate bonds and exchanges intracellular Na^+^ for extracellular K^+^ [73]. Inhibition of renal NKA in HW fish resulted in a reduced salt efflux in comparison with that of the control group. In addition, the Na^+^ influx enhanced the osmotic pressure of renal cells, which is required for adaptation to hypertonic environmental conditions. The Na^+^ influx resulted in an observed negative effect on DAT-mediated extracellular dopamine reuptake. In contrast, the increase in NKA activity in the FW group was accompanied by an increase in Na^+^ efflux, which induced extracellular dopamine clearance. These results indicated osmosis can be regulated by the renal dopamine system by altering dopamine release/reuptake and maintaining Na^+^ homeostasis during chronic salinity stress.

## Conclusions

**Fig. 9:**
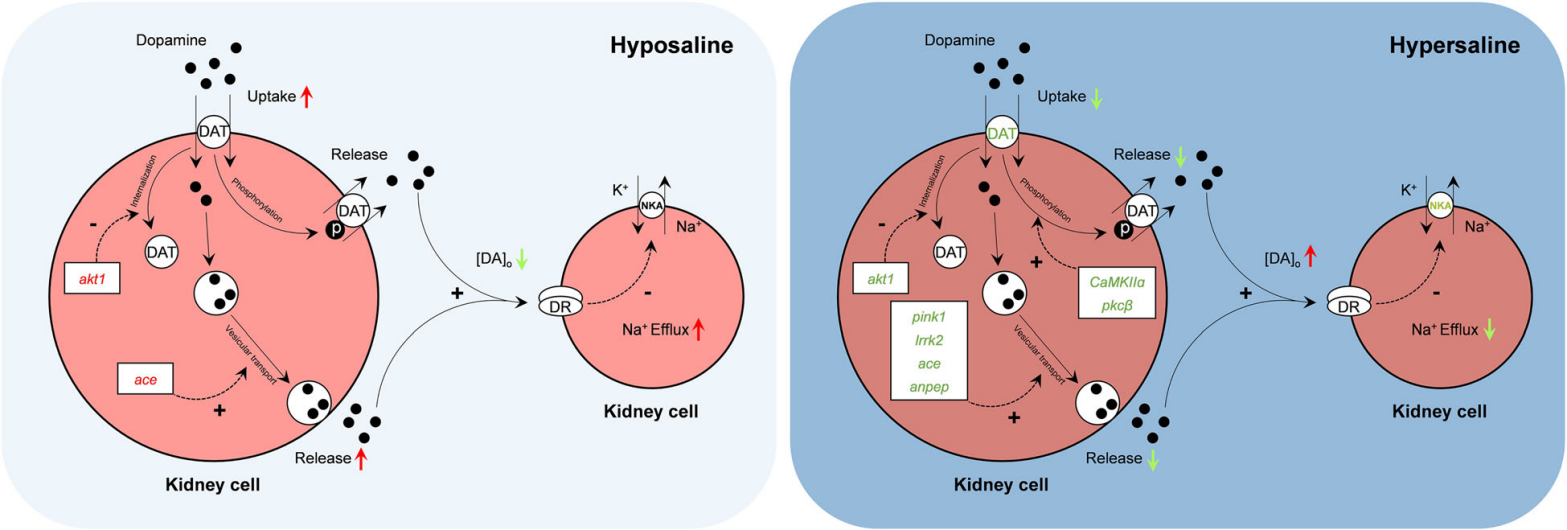
Effects of environmental salinity on dopamine transporter- and vesicular traffic-mediated dopamine release/reuptake and feedback regulation mechanisms of the renal dopamine system for Na^+^ homeostasis during chronic salinity stress

In the present study, a renal comparative transcriptomic study of *S. argus* exposed to different environmental salinities was conducted. Differences in transcriptomic levels under hyposaline and hypersaline conditions were revealed. The transcriptomic analyses indicated the renal dopamine system is essential in osmoregulation. The differential expression levels of dopamine release/reuptake-related genes, which ensured appropriate extracellular dopamine abundance, were reflected by changes in kidney function, including Na^+^ transport. The results of this study indicated the efficiency of extracellular dopamine clearance mediated by DAT was higher than that of dopamine release mediated by vesicular transport and phosphorylated DATs. Therefore, the concentration of extracellular dopamine decreased under hyposaline stress, which reduced the inhibition of NKA on the membrane, resulting in Na^+^ efflux. In contrast, increased dopamine levels were accompanied by the inhibition of NKA activity and expression, preventing the loss of Na^+^ (Fig. 9). In summary, this study showed renal dopamine directly affects Na^+^ homeostasis by interfering with dopamine transport during chronic salinity tolerance and provided new insights into the renal osmoregulation of marine fish.

## Additional files

Additional file 1: **Figure S1.** Length distribution of unigenes. Consensus sequence lengths ranged from 250 bp to greater than 2000 bp. The number of unigenes for each length range is indicated above each column. Unigenes with an average length of 250 bp were the most abundant (135,879), whereas unigenes with an average length of 1590 bp were the least abundant (2735). Sequences longer than 2000 bp were grouped together. The number of sequences decreased as length increased. (TIF 1027 kb)

Additional file 2: **Figure S2.** Characteristics of the homology search for Illumina sequences against the NCBI-NR database. Species distribution is shown as a percentage of the total homologous sequences with an E-value ≤1e-6. All proteins in the NCBI-NR database were used in the homology search. The best sequence hits were selected for analysis. (TIF 87320 kb)

Additional file 3: **Figure S3.** GO classification of unigenes. The results for ‘biological process (BP)’, ‘cellular component (CC)’ and ‘molecular function (MF)’ terms were summarized. (TIF 1925 kb)

Additional file 4: **Figure S4.** Eukaryotic orthologous group (KOG) classification of the assembled unigenes in *S. argus*. (TIF 22535 kb)

## Abbreviations

[DA]_i_: Intracellular dopamine level
[DA]_o_: Dopamine concentration in the culture medium
ACE: Angiotensin-converting enzyme
AKT1: RAC-alpha serine/threonine-protein kinase
Ang: angiotensin
APN: Aminopeptidase N
BP: Biological process
CaMKIIα: Calcium/calmodulin-dependent protein kinase II alpha
CC: Cellular component
cDNA: Complimentary DNA
Ct: Cycle threshold
DAT: Dopamine transporter
DEGs: Differentially expressed genes
FBS: Fetal bovine serum
FDR: False discovery rate
FITC: Fluorescein isothiocyanate
FPKM: Fragments per kilobase per transcript per million mapped reads
FW: Freshwater
GO: Gene Ontology
HRP: Horseradish peroxidase
HW: Hypersaline water
KEGG: Pathway Annotation of the Kyoto Encyclopedia of Genes and Genomes
KOG/COG: Clusters of Orthologous Groups of proteins
LRRK2: Leucine-rich repeat kinase 2
MF: Molecular function
NCBI: National Center for Biotechnology Information
NKA: Na^+^/K^+^-ATPase
NR: Non-redundant protein sequences
PBS: Phosphate buffer solution
PBST: PBS containing 0.5% Triton X-100
PINK1: PTEN-induced putative kinase 1;
PKCβ: Protein kinase C beta
PVDF: Polyvinylidene fluoride
qPCR: Quantitative PCR
RAS: renin-angiotensin system
RNA-Seq: high-throughput RNA sequencing
RT: Reverse transcription
SDS: Sodium dodecyl sulfate

## References

[1] Ong JJ, Rountrey AN, Meeuwig JJ, Newman SJ, Zinke J, Meekan MG (2015). Contrasting environmental drivers of adult and juvenile growth in a marine fish: implications for the effects of climate change. Sci Rep.

[2] Kalujnaia S, IS MW, Zaguinaiko VA, Feilen AL, Nicholson J, Hazon N (2007). Salinity adaptation and gene profiling analysis in the European eel (*Anguilla anguilla*) using microarray technology. Gen Comp Endocrinol.

[3] Tine M, de Lorgeril J, Panfili J, Diop K, Bonhomme F, Durand JD (2007). Growth hormone and Prolactin-1 gene transcription in natural populations of the black-chinned tilapia *Sarotherodon melanotheron* acclimatized to different salinities. Comp Biochem Physiol B Biochem Mol Biol.

[4] Silva PI, Martins CI, Höglund E, Gjøen HM, Øverli Ø (2014). Feeding motivation as a personality trait in Nile tilapia (*Oreochromis niloticus*): role of serotonergic neurotransmission. Fish Physiol Biochem.

[5] Ghazilou A, Chenary F, Morovvati H, Zolgarneine H (2011). Time course of saltwater adaptation in Spotted Scat (*Scatophagus argus*) (Pisces): A histomorphometric approach. Italian J Zool.

[6] Sivan G, Radhakrishnan CK (2011). Food, Feeding Habits and Biochemical Composition of *Scatophagus argus*. Turk J Fish Aquat Sci.

[7] Cieluch U, Charmantier G, Grousset E, Charmantier-Daures M, Anger K (2005). Osmoregulation, immunolocalization of Na^+^/K^+^-ATPase, and ultrastructure of branchial epithelia in the developing brown shrimp, *Crangon crangon* (*Decapoda, Caridea*). Physiol Biochem Zool.

[8] Hu P, Li S, Zhong Y, Mu X, Gui L, Zhang J (2014). Identification of *fxyd* genes from the spotted scat (*Scatophagus argus*): molecular cloning, tissue-specific expression, and response to acute hyposaline stress. Comp Biochem Physiol B Biochem Mol Biol.

[9] Mu X, Su M, Gui L, Liang X, Zhang P, Hu P, Liu Z, Zhang J (2015). Comparative renal gene expression in response to abrupt hypoosmotic shock in spotted scat (*Scatophagus argus*). Gen Comp Endocrinol.

[10] Su M, Mu X, Gui L, Zhang P, Zhou J, Ma J (2016). Dopamine regulates renal osmoregulation during hyposaline stress *via* DRD1 in the spotted scat (*Scatophagus argus*). Sci Rep.

[11] Marshall WS, Grosell M, Evans DH, Claiborne JB (2005). Ion transport, osmoregulation, and acid-base balance. The physiology of fishes.

[12] Taylor JR, Mager EM, Grosell M (2010). Basolateral NBCe1 plays a rate-limiting role in transepithelial intestinal HCO3^−^ secretion, contributing to marine fish osmoregulation. J Exp Bio.

[13] McCormick SD (2001). Endocrine control of osmoregulation in teleost fish. Am Zool.

[14] Evans DH, Piermarini PM, Choe KP (2005). The multifunctional fish gill: Dominant site of gas exchange, osmoregulation, acid-base regulation, and excretion of nitrogenous waste. Physiol Rev.

[15] Brijs J, Axelsson M, Gräns A, Pichaud N, Olsson C, Sandblom E (2015). Increased gastrointestinal blood flow: An essential circulatory modification for euryhaline rainbow trout (*Oncorhynchus mykiss*) migrating to sea. Sci Rep.

[16] Perry SF, Shahsavarani A, Georgalis T, Bayaa M, Furimsky M, Thomas SL (2003). Channels, pumps, and exchangers in the gill and kidney of freshwater fishes: their role in ionic and acid-base regulation. J Exp Zool A Comp Exp Biol.

[17] Jiang X, Chen W, Liu X, Wang Z, Liu Y, Felder RA (2016). The Synergistic roles of cholecystokinin B and dopmine D5 receptors on the regulation of renal sodium excretion. PLoS One.

[18] Westphal A, Mrowka R (2018). New insights into the astonishing diversity of hormone functions. Acta Physiol.

[19] McCormick SD, Bradshaw D (2006). Hormonal control of salt and water balance in vertebrates. Gen Comp Endocrinol.

[20] Mesey E, Eisenhofer G, Harta G, Hansson S, Gould L, Hunyady B, Hoffman BJ (1996). A novel nonneuronal catecholaminergic system: Exocrine pancreas synthesizes and releases dopamine. Proc Natl Acad Sci USA.

[21] Felder RA, Jose PA (2006). Mechanisms of disease: the role of GRK4 in the etiology of essential hypertension and salt sensitivity. Nat Clin Pract Nephrol.

[22] Likhite N, Jackson CA, Liang MS, Krzyzanowski MC, Lei P, Wood JF (2015). The protein arginine methyltransferase PRMT5 promotes D_2_-like dopamine receptor signaling. Sci Signal.

[23] Felder RA, Sanada H, Xu J, Yu PY, Wang Z, Watanabe H (2002). G protein-coupled receptor kinase 4 gene variants in human essential hypertension. Proc Natl Acad Sci USA.

[24] Natarajan AR, Eisner GM, Armando I, Browning S, Pezzullo JC, Rhee L (2016). The Renin-Angiotensin and Renal Dopaminergic Systems Interact in Normotensive Humans. J Am Soc Nephrol.

[25] Fiol DF, Kultz D (2007). Osmotic stress sensing and signaling in fishes. FEBS J..

[26] Wang Z, Gerstein M, Snyder M (2009). RNA-Seq: a revolutionary tool for transcriptomics. Nat Rev Genet.

[27] Oshlack A, Robinson MD, Young MD (2010). From RNA-seq reads to differential expression results. Genome Biol.

[28] Tarazona S, García-Alcalde F, Dopazo J, Ferrer A, Conesa A (2011). Differential expression in RNA-seq: a matter of depth. Genome Res.

[29] Bolger AM, Lohse M, Usadel B (2014). Trimmomatic: A flexible trimmer for Illumina Sequence Data. Bioinformatics.

[30] Grabherr MG, Haas BJ, Yassour M, Levin JZ, Thompson DA, Amit I (2011). Full-length transcriptome assembly from RNA-Seq data without a reference genome. Nat Biotechnol.

[31] Langmead B, Trapnell C, Pop M, Salzberg SL (2009). Ultrafast and memory-efficient alignment of short DNA sequences to the human genome. Genome Biol.

[32] Li B, Dewey CN (2011). RSEM: accurate transcript quantification from RNA-Seq data with or without a reference genome. BMC Bioinformatics.

[33] Li H, Handsaker B, Wysoker A, Fennell T, Ruan J, Homer N (2009). The Sequence Alignment/Map format and SAMtools. Bioinformatics.

[34] Li H, Durbin R (2010). Fast and accurate long-read alignment with Burrows-Wheeler transform. Bioinformatics.

[35] Altschul SF, Madden TL, Schäffer AA, Zhang J, Zhang Z, Miller W (1997). Gapped BLAST and PSI-BLAST: a new generation of protein database search programs. Nucleic Acids Res.

[36] Conesa A, Götz S, García-Gómez JM, Terol J, Talón M, Robles M (2005). Blast2GO: A universal tool for annotation, visualization and analysis in functional genomics research. Bioinformatics.

[37] Mao X, Cai T, Olyarchuk JG, Wei L (2005). Automated genome annotation and pathway identification using the KEGG Orthology (KO) as a controlled vocabulary. Bioinformatics.

[38] Robinson MD, McCarthy DJ, Smyth GK (2010). edgeR: a Bioconductor package for differential expression analysis of digital gene expression data. Bioinformatics.

[39] Pridgeon JW, Shoemaker CA, Klesius PH (2010). Identification and expression profile of multiple genes in the anterior kidney of channel catfish induced by modified live *Edwardsiella ictaluri* vaccination. Vet Immunol Immunopathol.

[40] Pfaffl MW (2001). A new mathematical model for relative quantification in real-time RT-PCR. Nucleic Acids Res.

[41] Lakra WS, Goswami M, Rajaswaminathan T, Rathore G (2010). Development and characterization of two new cell lines from common carp, *Cyprinus carpio* (Linn). Biol Res.

[42] Gene Ontology C (2008). The Gene Ontology project in 2008. Nucleic Acids Res..

[43] Tatusov RL, Fedorova ND, Jackson JD, Jacobs AR, Kiryutin B, Koonin EV (2003). The COG database: an updated version includes eukaryotes. BMC Bioinformatics..

[44] Kanehisa M, Araki M, Goto S, Hattori M, Hirakawa M, Itoh M, Katayama T, Kawashima S, Okuda S, Tokimatsu T (2008). KEGG for linking genomes to life and the environment. Nucleic Acids Res..

[45] Kulczykowska E (2001). A review of the multifunctional hormone melatonin and a new hypothesis involving osmoregulation. Rev Fish Biol Fish..

[46] Hoshijima K, Hirose S (2007). Expression of endocrine genes in zebrafish larvae in response to environmental salinity. J Endocrinol.

[47] Seale AP, Watanabe S, Breves JP, Lerner DT, Kaneko T, Gordon Grau E (2012). Differential regulation of TRPV4 mRNA levels by acclimation salinity and extracellular osmolality in euryhaline tilapia. Gen Comp Endocrinol.

[48] Jose PA, Soares-da-Silva P, Eisner GM, Felder RA (2010). Dopamine and G protein-coupled receptor kinase 4 in the kidney: role in blood pressure regulation. Biochim Biophys Acta.

[49] Richardson BD, Saha K, Krout D, Cabrera E, Felts B, Henry LK (2016). Membrane potential shapes regulation of dopamine transporter trafficking at the plasma membrane. Nat Commun..

[50] Garcia BG, Wei Y, Moron JA, Lin RZ, Javitch JA, Galli A (2005). Akt is essential for insulin modulation of amphetamine-induced human dopamine transporter cell-surface redistribution. Mol Pharmacol.

[51] Torres GE, Gainetdinov RR, Caron MG (2003). Plasma membrane monoamine transporters: structure, regulation and function. Nat Rev Neurosci..

[52] Raiteri L, Raiteri M (2015). Multiple functions of neuronal plasma membrane neurotransmitter transporters. Prog Neurobiol..

[53] Matta S, Van Kolen K, da Cunha R, van den Bogaart G, Mandemakers W, Miskiewicz K (2012). LRRK2 controls an EndoA phosphorylation cycle in synaptic endocytosis. Neuron..

[54] Esposito G, Ana Clara F, Verstreken P (2012). Synaptic vesicle trafficking and Parkinson's disease. Dev Neurobiol..

[55] Martin I, Kim JW, Lee BD, Kang HC, Xu JC, Jia H (2014). Ribosomal protein s15 phosphorylation mediates LRRK2 neurodegeneration in Parkinson's disease. Cell.

[56] Parisiadou L, Yu J, Sgobio C, Xie C, Liu G, Sun L (2014). LRRK2 regulates synaptogenesis and dopamine receptor activation through modulation of PKA activity. Nat Neurosci..

[57] Schwab AJ, Ebert AD (2015). Neurite Aggregation and Calcium Dysfunction in iPSC-Derived Sensory Neurons with Parkinson's Disease-Related LRRK2 G2019S Mutation. Stem Cell Rep..

[58] Li X, Patel JC, Wang J, Avshalumov MV, Nicholson C, Buxbaum JD (2010). Enhanced striatal dopamine transmission and motor performance with LRRK2 overexpression in mice is eliminated by familial Parkinson's disease mutation G2019S. J Neurosci..

[59] Piccoli G, Condliffe SB, Bauer M, Giesert F, Boldt K, De Astis S (2011). LRRK2 controls synaptic vesicle storage and mobilization within the recycling pool. J Neurosci..

[60] Knott AB, Perkins G, Schwarzenbacher R, Bossy-Wetzel E (2008). Mitochondrial fragmentation in neurodegeneration. Nat Rev Neurosci..

[61] Rappold PM, Cui M, Grima JC, Fan RZ, de Mesy-Bentley KL, Chen L (2014). Drp1 inhibition attenuates neurotoxicity and dopamine release deficits *in vivo*. Nat Commun.

[62] Kitada T, Pisani A, Porter DR, Yamaguchi H, Tscherter A, Martella G (2007). Impaired dopamine release and synaptic plasticity in the striatum of PINK1-deficient mice. Proc Natl Acad Sci USA..

[63] Sun J, Kouranova E, Cui X, Mach RH, Xu J (2013). Regulation of dopamine presynaptic markers and receptors in the striatum of DJ-1 and Pink1 knockout rats. Neurosci Lett.

[64] Labandeira-García JL, Garrido-Gil P, Rodriguez-Pallares J, Valenzuela R, Borrajo A, Rodríguez-Perez AI (2014). Brain renin-angiotensin system and dopaminergic cell vulnerability. Front Neuroanat.

[65] Firouzabadi N, Ghazanfari N, Alavi Shoushtari A, Erfani N, Fathi F, Bazrafkan M (2016). Genetic Variants of Angiotensin-Converting Enzyme Are Linked to Autism: A Case-Control Study. PLoS One.

[66] Hui L, Wu JQ, Zhang X, Lv J, Du WL, Kou CG (2014). Association between the angiotensin-converting enzyme gene insertion/deletion polymorphism and first-episode patients with schizophrenia in a Chinese Han population. Hum Psychopharmacol.

[67] Neasta J, Valmalle C, Coyne AC, Carnazzi E, Subra G, Galleyrand JC (2016). The novel nonapeptide acein targets angiotensin converting enzyme in the brain and induces dopamine release. Br J Pharmacol.

[68] Carey RM, Padia SH (2013). Role of angiotensin AT(2) receptors in natriuresis: Intrarenal mechanisms and therapeutic potential. Clin Exp Pharmacol Physiol.

[69] Stragier B, Demaegdt H, De Bundel D, Smolders I, Sarre S, Vauquelin G (2007). Involvement of insulin-regulated aminopeptidase and/or aminopeptidase N in the angiotensin IV-induced effect on dopamine release in the striatum of the rat. Brain Res.

[70] Namkung Y, Sibley DR (2004). Protein kinase C mediates phosphorylation, desensitization, and trafficking of the D2 dopamine receptor. J Biol Chem.

[71] Luderman KD, Chen R, Ferris MJ, Jones SR, Gnegy ME (2015). Protein kinase C beta regulates the D_2_-like dopamine autoreceptor. Neuropharmacology.

[72] Sulzer D, Sonders MS, Poulsen NW, Galli A (2005). Mechanisms of neurotransmitter release by amphetamines: a review. Prog Neurobiol.

[73] Zhang LN, Sun YJ, Wang LX, Gao ZB (2016). Glutamate Transporters/Na(+), K(+)-ATPase Involving in the Neuroprotective Effect as a Potential Regulatory Target of Glutamate Uptake. Mol Neurobiol.

[74] Chinigarzadeh A, Muniandy S, Salleh N (2015). Estrogen, progesterone, and genistein differentially regulate levels of expression of α-, β-, and γ-epithelial sodium channel (ENaC) and α-sodium potassium pump (Na^+^/K^+^-ATPase) in the uteri of sex steroid-deficient rats. Theriogenology.

[75] Giros B, Caron MG (1993). Molecular characterization of the dopamine transporter. Trends Pharmacol Sci.

