# Supplementary figures and images for: Transcriptional analysis of renal dopamine-mediated Na+ homeostasis response to environmental salinity stress in Scatophagus argus
Source: BMC Genomics. 2019 May 24;20:418. doi: 10.1186/s12864-019-5795-x (PMC6534869; doi:10.1186/s12864-019-5795-x)

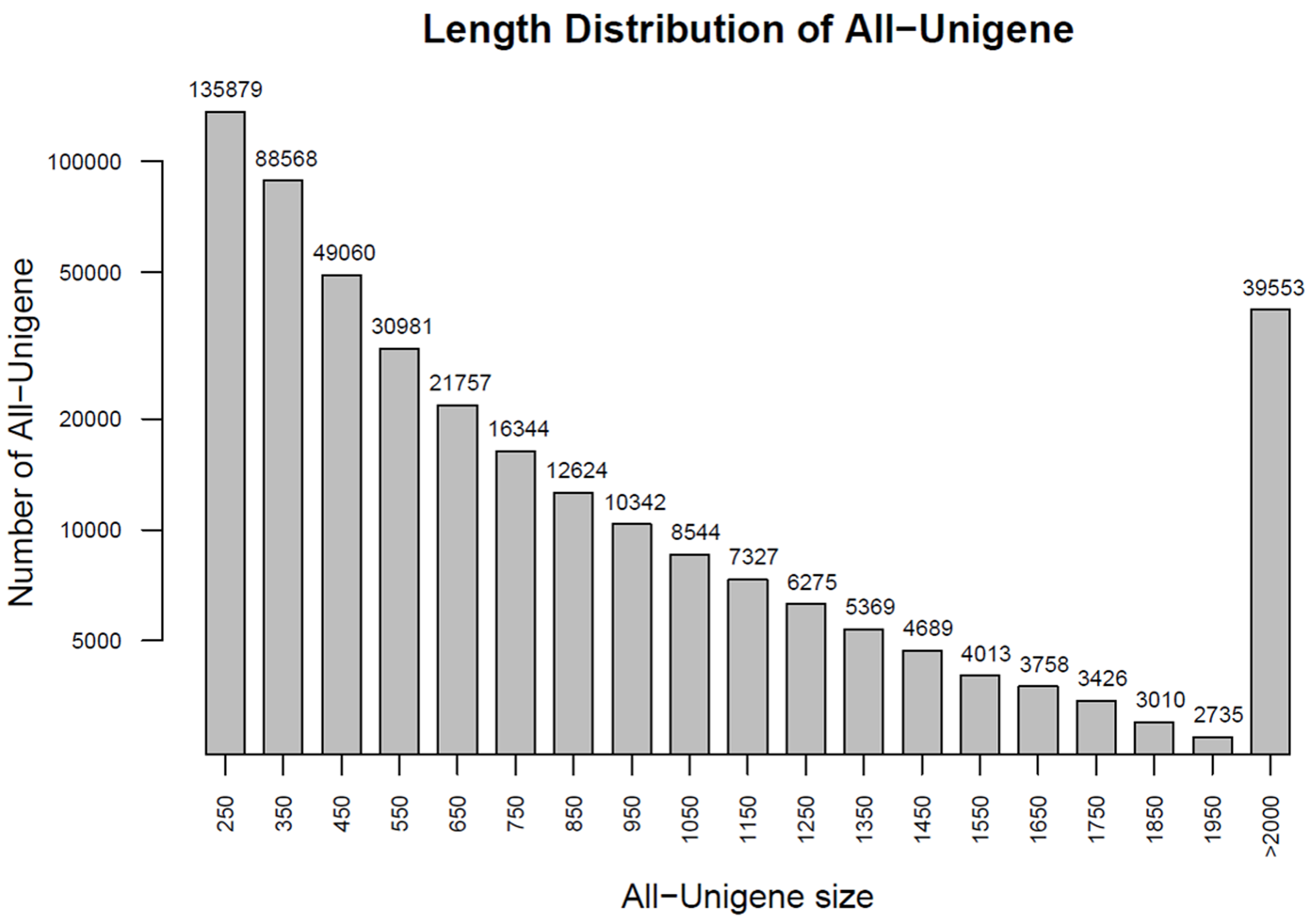

Supplement: Supplementary file 1 — Figure S1. Length distribution of unigenes. Consensus sequence lengths ranged from 250 bp to greater than 2000 bp. The number of unigenes for each length range is indicated above each column. Unigenes with an average length of 250 bp were the most abundant (135,879), whereas unigenes with an average length of 1590 bp were the least abundant (2735). Sequences longer than 2000 bp were grouped together. The number of sequences decreased as length increased. (TIF 1027 kb) [file 12864_2019_5795_MOESM1_ESM.tif]

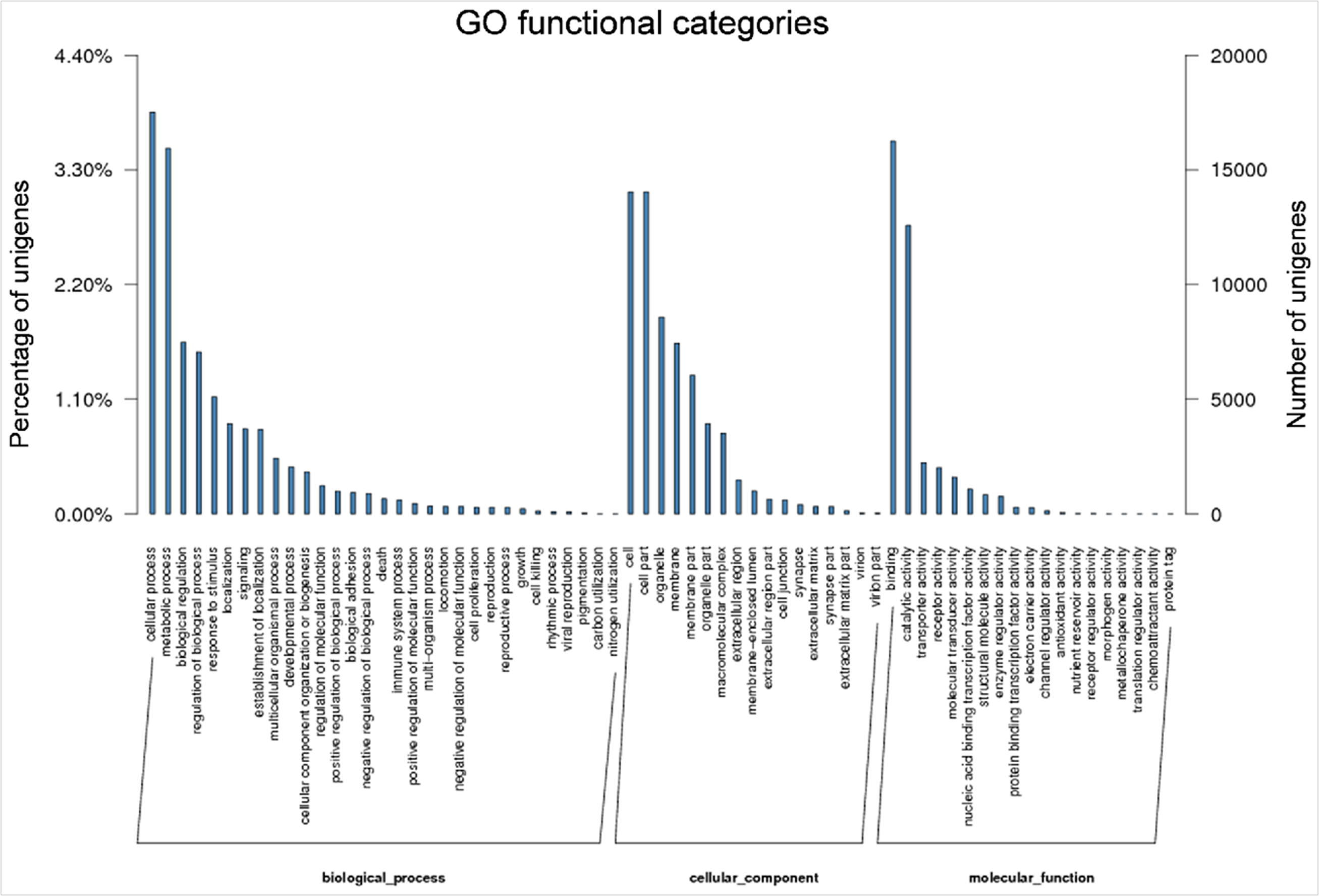

Supplement: Supplementary file 3 — Figure S3. GO classification of unigenes. The results for ‘biological process (BP)’, ‘cellular component (CC)’ and ‘molecular function (MF)’ terms were summarized. (TIF 1925 kb) [file 12864_2019_5795_MOESM3_ESM.tif]
